# Age in the Time of COVID-19: An Ethical Analysis

**DOI:** 10.14336/AD.2020.0929

**Published:** 2021-02-01

**Authors:** Sorin Hostiuc, Ionut Negoi, Oana Maria-Isailă, Ioana Diaconescu, Mihaela Hostiuc, Eduard Drima

**Affiliations:** ^1^Carol Davila University of Medicine and Pharmacy, Bucharest, Romania; ^2^National Institute of Legal Medicine, Bucharest, Romania; ^3^Floreasca Clinical Emergency Hospital, Bucharest, Romania; ^4^Dunărea de Jos University, Galați, Romania

**Keywords:** age, morality, COVID-19, pandemic, medical futility

## Abstract

Despite using a myriad of methods to combat the spread of COVID-19, the healthcare systems (especially the intensive care units) have been overwhelmed, showing an outpaced capacity of available beds and ventilators. Choosing the right criteria to allocate the scarce ICU seems very challenging, being necessary a rapid, uncomplicated and universally accepted tool for patients’ triage regarding access to lifesaving resources; one such criterion, which generates intense debates, is age. Under certain circumstances, it might seem appropriate to choose to treat a young over an old patient. The main advantage of this approach is the potential for long-term survival, implying an equal right to reach an advanced age. Many authors have given moral reasons to support it, mainly based on utilitarian ethics or on distributive justice. However, there are numerous counterarguments to this approach, which we will summarize in this article. We will show that age should never be used as a unique criterion for withholding/not initiating life-saving procedures, even in pandemics or cases in which healthcare resources are extremely scarce. This approach is based on fundamental Codes of Ethics, such as the WMA Code of Ethics or the Oath of Hippocrates and all physicians treating patients should obey them.

Coronavirus disease has generated a significant burden on society in general and on healthcare systems in particular, both through an increased number of cases and fatalities but also through substantial socio-economic consequences. To limit the spread of the COVID-19 disease, various methods have been used, with variable success from country to country, including social distancing, increased population testing, border closing, and so on. Despite these measures, in many countries, the healthcare systems have been overwhelmed, and the number of patients requiring specialized medical care, and especially intensive care, is well above the number of available beds or ventilators [1-3]. Italy has been a prime example in this regard, with the number of patients (especially those requiring access to ventilators) rapidly outpacing the available number of beds (respirators), leading to difficult choices regarding who to treat. Lisa Rosenbaum gave a clear example in this regard: “Dr. S. offered a hypothetical scenario involving two patients with respiratory failure, one 65 and the other 85 with coexisting conditions. With only one ventilator, you intubate the 65-year-old. Dr. D. told me his hospital was also considering, in addition to the number of comorbidities, the severity of respiratory failure and probability of surviving prolonged intubation, aiming to dedicate its limited resources to those who both stand to benefit most and have the highest chance of surviving” [4]. Società Italiana di Anestesia Analgesia Rianimazione e Terapia Intensiva (SIIAARTI) made, in its “Clinical Ethics Recommendations for the Allocation of Intensive Care Treatments in Exceptional, Resource-Limited Circumstances” [5], a few controversial recommendations, such as: “As an extension of the principle of proportionality of care, allocation in a context of serious shortage of healthcare resources, we must aim at guaranteeing intensive treatments to patients with greater chances of therapeutic success. Therefore, it is a matter of favoring the “greatest life expectancy”. The need for intensive care must be integrated with other elements of “clinical suitability”, thus including: the type and severity of the disease, the presence of comorbidities, the impairment of other organs and systems, and their reversibility. This means, not necessarily having to follow a criterion for access to intensive care like “first come, first served”, Together with age, the comorbidities and functional status of any critically ill patient presenting in these exceptional circumstances should carefully be evaluated. A longer and, hence, more “resource consuming” clinical course may be anticipated in frail elderly patients with severe comorbidities, as compared to a relatively shorter, and potentially more benign course in healthy young subjects” and especially “An age limit for the admission to the ICU may ultimately need to be set. The underlying principle would be to save limited resources which may become extremely scarce for those who have a much greater probability of survival and life expectancy, in order to maximize the benefits for the largest number of people. In the worst-case scenario of complete saturation of ICU resources, keeping a “first come, first served” criterion would ultimately result in withholding ICU care by limiting ICU admission for any subsequently presenting patient” [5]. In the UK, the British Medical Association, in the “COVID-19 - ethical issues. A guidance note” states: “During the peak of the pandemic, it is possible that doctors will be required to assess a person’s eligibility for treatment based on a ‘capacity to benefit quickly’ basis. As such, some of the most unwell patients may be denied access to treatment such as intensive care or artificial ventilation. This will inevitably have a disproportionate impact on older people and those with long-term health conditions that have a direct bearing on their ability to recover quickly” [6]. In Mexic, an initial version of the guidelines regarding the allocation of medical resources in a contingency situation said that it would prioritize young over older adults; this recommendation has been eliminated in subsequent versions [7].

Other countries have implemented alternative allocations algorithms. For example, the American Geriatric Society position was that age should be avoided as a means for excluding anyone from care, and states specifically that “allocation frameworks that use age as a categorical exclusion violate this provision of federal antidiscrimination law” [8]. The Canadian Geriatric Society also states that “age alone should not drive decisions for health-care resource allocation” in this pandemic [9].

The purpose of this article is to evaluate whether using age as a sole criterion for resource allocation in the COVID-19 pandemic is morally justifiable.

## Age discrimination in resource allocation

Rationalizing health care based on age has been repeatedly proposed by many authors [10]; based on this theory, if a person reaches a certain age (usually above 80), the efforts of the healthcare system should be directed toward palliative treatments or increasing the quality of life [11-13]. Moreover, the general public in many countries seems to prioritize the young. A study from Sweden, for example, showed that, as an average, people were willing to sacrifice thirty-five seventy-year-olds to save one thirty-years-old [14]. Another study, which was performed in the US, aimed to evaluate the response to pandemic similar to the Spanish flu from 1918. The authors have found that “although age should be neither the primary nor sole criterion for resource allocation in disaster contexts, there are circumstances in which it may be appropriate to consider stage of life in decision-making. For these reasons, in the event that other criteria result in equivalent priority scores, equal and highest priority in this system is given to children and to adults age 49, whose death is most likely to impose hardship on other people whose well-being depends directly on their support (children, elderly relatives). Adults who have not yet lived a full life (age 50-69) are given next priority, followed by those who are approaching or have reached the high end of average life expectancy (age 70-84). Lowest priority is given to patients 85 and older”[15]. Bowling, using a representative sample from Great Britain, has shown that subjects tended to attribute the highest priority to treatments of children with threatening illnesses, and the lowest priority to treatments for infertility and treatments for people aged over 75 with life-threatening illnesses [16].

The main reason for this approach is the potential for long-term survival, implying an equal right to reach an advanced age. Briefly, this means that everybody should be allowed to become old, and those who already reached this stage of their life should be deprioritized from healthcare measures aimed to increase the lifespan, if other, younger persons need the same medical resources [15]. This approach is not necessarily an unjustifiable one, and many authors have given moral reasons to support it, mainly based on utilitarian ethics or on distributive justice. Nilstun and Ohlsson for example, have argued that, even if healthcare discrimination by age should, as a general rule, be avoided, based on the principles of equality and solidarity, it could be morally justified, in situations with intrinsic scarcity (such as transplantation and, we might add - pandemics), based on the principles of solidarity and efficiency [17]. Lambie has debated, based on Norman Daniels’ “Prudential Lifespan Account” [18] that age prioritization (another wording for age discrimination) can be allowed if applied consistently over the lifespan of all members of a society; this approach will enable a fairer distribution of the resources between generations and a normal range of opportunity for everybody [19].

Another argument favoring the discrimination of the elderly, which has appeared from time to time in the literature, is represented by the fact that they are sometimes willing to be deprioritized when the resources are scarce [10, 20]. Such an example has recently reached the media outlets - an elderly priest (Don Guiseppe Berardelli), had requested to be removed from the ventilator, to allow its use to treat a younger patient (USA Today). If they are willing (and therefore make this decision autonomously), why not let them?

Cesare and Proietti have argued that, in the context in the COVID-19 pandemic, physicians need robust and transparent criteria to make fast-paced decisions and, as other more robust parameters than age have not yet been identified, its use can be morally justified: “We must show that we understand why intensive care physicians are prioritizing the life of a 40-year-old person over that of a 90 year-old, and that this is the best decision. They have never been exposed to anything other than this approach. And the critical nature of the situation can further provide ground for justifying such arguable choices. “All is fair in love and war”—and we are indeed in war!” [21].

## Arguments against using age discrimination for resource allocation in the elderly

There are extremely numerous counterarguments to age discrimination in resource allocation in the elderly, of which only a few, more relevant to the context of pandemics, will be discussed here.

Ageism, according to Robert Butler, who coined the term in 1968, in a Washington Post interview is the process of systematic sterotyping of and discrimination against people because they are old [22]. In healthcare, ageism is often present, even if not always obvious. To delink age discrimination from ageism, Savulescu *et al* argued that “age is thus a de facto measure of length. Because older people tend to die sooner than younger people, utilitarianism tends to favor saving the lives of the younger. However, age itself does not matter; it is the expected length of the benefit. This is why utilitarianism is not unfairly discriminatory, and not “ageist” in an ethically problematic sense” [23]. Sometimes, the physician does not pay the same attention to a disease with a similar severity in the elderly as in a younger patient, many organic symptoms are attributed to neurologic or psychiatric impairments, screening procedures are recommended less often, surgery and other invasive treatments are recommended and performed less often without an objective proof they are futile, based on subjective criteria such decreased physical strength, decreased therapeutic compliance, a less significant increase in survival or disease-free survival, *etc*. [24-27] The fatality rate of COVID-19 is highly dependent on age. Onder *et al.* showed, for example, a fatality rate of 0.2-0.3% in the age group 30-39, 3.5-3.6% in the age group 60-69, and 14.8-20.2% in the age group above 80 [28]. However, this is not the only significant risk factor for fatality associated with this disease, as male gender, hypertension, diabetes, coronary heart disease, chronic obstructive lung disease, and other conditions being also associated with increased mortality[29]. The elderly tends to have an increased number of comorbidities significantly associated with increased mortality, and these may be, in part at least, the reason for the positive association between age and fatality. However, let us look at another demographic risk factor - gender. According to Zhou *et al.* in the non-survivor group of COVID-19 patients, the male:female ratio was 70:30, while in the survivor group, the ratio was 59:41. Males are more prone to a series of COVID-19 comorbidities such as hypertension, diabetes, coronary heart disease, smoking, *etc.* (at least in some regions) [29]. Why not use gender to make decisions regarding who should use the ventilator? It is a criterion at least as robust as age [21]. Moreover, there are reports of persons over 100-years-old surviving COVID-19 infection, including a 103-years-old woman who also survived the Spanish flu (as reported by Sky News), suggesting that even the very elderly can survive it, and that treatments are efficient in this age group. Once a therapeutic alliance has been established, physicians should not make clinical decisions regarding who to treat based on statistics, but rather treat everybody who has a medical indication, even if the odds are against a favorable course of the disease. If, however, there is no clear-cut medical indication for a certain procedure, it should not be performed. Statistics should only be used to provide objective, evidence-based criteria based on which to establish medical indications. If the treatment is efficient, why de-prioritize a particular age group? If we are to extrapolate this approach, we might say, for example, that transplant should be reserved only for the young (which will most likely remove many bottlenecks in transplantology)[30].

There is an increased difficulty in handling new diseases, where the indications for therapy are not yet established, as is the case with COVID-19, in which there was no time to develop and validate prognostic scores, based on years of life lost, or years lived with disability. When a medical indication based on established guidelines and prognostic scores is not yet available, physicians should have more leniency in establishing it at the bedside, based on a mix of science (what available data is present) and art (professional experience, the professional “feeling” of the highly skilled physician who has seen thousands, or tens of thousands of cases in his professional lifetime). This means that they can choose who and how to treat, depending on the particularities of the case. For example, the physician may consider that a person with multiple comorbidities, who has a very slim chance of survival, does not have a medical indication for potentially curable treatment when this is scarce, and there are other patients, with much higher probabilities of survival. That patient should, however, receive the maximum degree of medical care that is available, to prolong his life and to alleviate his suffering. This approach is in line with WMA Code of Ethics, stating that “a physician shall strive to use health care resources in the best way to benefit patients and their community” [31].

There is a highly relevant distinction that has to be made when discussing ageism in resource allocation, between age discrimination per se and evidence-based medical decisions in which age is a relevant factor. There are numerous clinical situations in which there is a significant correlation between age and prognosis, or health risk, or the efficacy of a therapeutic intervention. For example, in the context of an influenza outbreak, if the mortality is significantly higher in the elderly, they should be prioritized in vaccination [32]. Similarly, if small children, in the same context, do not respond to antiviral therapy, they should not receive it [32]. Collet et al showed that the use of levothyroxine in patients above 65 with subclinical hypothyroidism do not improve Hypothyroid Symptoms, and Tiredness Score, and do not improve blood pressure, cognition, weight or muscle strength [33], making this drug inefficient in this age group. These are not examples of age discrimination, but rather of medical decisions in which age is a relevant clinical factor. In the context of the COVID-19 pandemic, there is no relevant data suggesting that treatment is inefficient in the elderly; they indeed need more time on the ventilator (their recovery is slower), and the mortality is indeed higher. But these are not clinically relevant criteria to deprioritize the elderly, but rather particularities of the therapy and prognosis in this age group. The WMA Code of Ethics states, also, that “A physician shall always bear in mind the obligation to respect human life” and that “a physician shall owe his/her patients complete loyalty and all the scientific resources available to him/her” [31]. How can these be respected when a person needing a ventilator is taken out of it based solely on its age? Physicians, once they have accepted a patient (and therefore have established a civil, contractual relationship with him/her), have a series of absolute duties directed specifically toward him/her, including the duties of diligence, loyalty or beneficence. These duties should be balanced with resource allocation criteria, which can be used in triage [34], before a physician-patient relationship is established. Afterward, the physician should always prioritize his/her patient over others, while respecting the need to allocate resources in a manner that should not be maleficent to other patients or the society at large. Decisions regarding resource allocation should not be made, as a general rule, by the treating-physicians, unless they are based on clinically relevant criteria, and only with a minimal of subjectivity. They must understand that they cannot always treat everybody, they cannot be judges of who lives and who dies, but only be providers of healthcare to as many in need as possible, while maintaining their professional and moral integrity. If the resources are scarce, as it happens almost everywhere in the context of the COVID-19 pandemics, prioritization of patients should only be based on clinically relevant, non-discriminatory criteria. Age as a sole criterion is not, based on current information regarding this disease, an objective criterion not to initiate certain therapies, but rather an element potentially increasing the gravity of the disease and the mortality, similar to gender, hypertension, diabetes, ischemic cardiac disease, and so on. Elderly patients should receive an optimum level of care within the limits of the available resources.

As seen above, there are studies showing that the general public tends to prefer a reallocation of scarce resources toward the young (all other conditions being the same). However, medicine is not a democracy - it is not the majority that decides who should get treated, but rather scientific evidence and physicians should not act as politicians, to reach high approval rates, but rather like professionals, who are bound by specific duties and moral codes, transcending the layperson view. Physicians should base their practice and decisions on the recommendations of professional societies and national guidelines, within the limits of their professional ethics; the opinions of some physicians or, even more, bioethicists, should not be regarded as justifications for unethical measures. Just because we see an interview or read an article in which a physician does something, or a bioethicist or a lawmaker argues something, this does not mean that we should take that information as an absolute (or even as a relative truth). We should always filter that information through our moral conscience before applying it to our patients. We, as physicians, are bound by fundamental ethical principles that define our moral behavior and our responsibility toward our patients, and the society in general. These principles are of two main types - depending on the moral philosophy we feel represents us (such a Kantian theory, utilitarianism, virtue ethics, prima facie ethics), namely general (and here a prime example is the principlist theory of Beauchamp and Childres [35]), and specific, or professional ethical principles (Professional Codes of Ethics). The principles of professional ethics should be respected by everybody; they are usually enforced at a national level, through guild-like organizations (like Medical College Boards); they are based on general principles of ethics and international codes of ethics (such as the WMA that was quoted here), and usually have a dominant moral philosophy beneath (usually Kantian or utilitarian). The recommendation of professional structures (such as medical societies) should always be filtered through these moral guidelines. In “times of war”, as this pandemic has been described, these general ethical guidelines still stand and should be applied by every physician, at the best of his/her knowledge.

We might also look at this issue of differential resource allocation based on age from a different perspective - who has higher chances to survive, even without ICU? A young person with fewer comorbidities, or an elderly with multiple comorbidities? If the young person, having fewer comorbidities, has increased chances of surviving, even without a respirator, why not prioritize an older person, who has fewer chances of surviving without it? The increased mortality in the elderly in COVID-19 may be explained, at least partially, by a discretionary allocation of resources toward the young.

Another argument supporting the prioritization of resources toward the young has been represented by the willingness of elderly patients to “take a back seat” [20]. This approach might be caused by them internalizing some arguments about the prioritization of the younger patients, to which they do not actually relate to [10, 20]. The elderly have a decreased internal resistance to external controls [35, 36], and subsequently are more prone to be influenced by arguments regarding age-dependent health-care prioritization, and may even act based on these arguments, even if they do not intrinsically agree with them. If this is the case, the voluntariness of a selfless act from an elderly might be altered, and his/her decisional capacity would subsequently be impaired. Of course, the evaluation of external controls acting outside the therapeutic alliance is beyond the control of the physician; however, he/she should take precautions when discussing with the patient in order not to increase, voluntarily or involuntarily, the magnitude of these external controls. This altruistic attitude is not present in practice as much as it has been hypothesized [10], and a public attitude should not, as stated above, be used to develop treatment selection algorithms by the physicians, especially in emergencies. If an elderly patient asks the physician to reallocate the medical resources used on him/her to another patient, the physician can and should respect the wish of the patient (provided he/she has decisional capacity).

Some authors argue that benefit maximization is paramount in epidemics, irrespective of the individual benefits for the patients. Emanuel et al said, for example, that “Because maximizing benefits is paramount in a pandemic, we believe that removing a patient from a ventilator or an ICU bed to provide it to others in need is also justifiable and that patients should be made aware of this possibility at admission... many guidelines agree that the decision to withdraw a scarce resource to save others is not an act of killing and does not require the patient’s consent” [37]. We believe this to be a slippery-slope argument - if removing someone from the ventilator to save another person is not killing, what other procedures aimed to save patients with higher chances of survival should also be allowed? For example, organ shortage is a known problem in transplantology. Why not use the elderly, or some other group with slim chances of survival to remove their organs before they are dead? After all, the dead donor rule has been under intense scrutiny in the last decades [38, 39]. This theoretical approach would decrease the trust in the medical profession, with potential consequences that would, most likely endure after the end of this pandemic, with decreased addressability toward healthcare specialists. Why would a patient trust the physician who, in certain circumstances, could stop a life-saving procedure, without his/her consent? Why should he, if sick, go to the hospital and not rather die at home? On the long term, from an utilitarian point of view, we believe that such an approach would increase overall morbidity and mortality, and would lead to less social benefit, compared to saving some patients, by removing others from the ventilators, without their consent.

Of course, it may seem easy to say what a physician should not do, but not give practical recommendations on what to do, and we will not make this mistake. The first thing any physician should do is to understand that, once he/she accepts a patient, he has certain moral duties toward him/her, including loyalty, fidelity, duty to treat, duty to respect his autonomy, duty to do no harm. Accepting a patient should not be dependent upon potentially discriminatory criteria (such as age, or gender, or race). How to treat the patient, when there are no established guidelines, should be left at the disposal of the physician, who should establish beforehand the types of treatment that could be potentially beneficial to the patient, and inform him correspondingly (or his legal representative, if the patient does not have decisional capacity). If the physician decides that a patient does not have an indication for an invasive treatment (due to its biological frailty, or the futility of the treatment), he/she has to inform the patient when the therapeutic alliance is initiated, or when the futility of the treatment becomes obvious. The physicians should, however, make sure that she/he gives equal treatment to equally sick patients, namely that he/she does not establish the medical indication for a treatment depending on irrelevant medical criteria (such as profession or wealth). If the number of patients needing specialized care in triage is greater than the available capabilities of the healthcare institution, random selection is preferable instead of selecting only a few patients from a group [37] based on irrelevant moral differences such as age or gender.

However, once the physician has started a certain treatment, she/he cannot stop it and give it to another, just because somebody else needs it more (unless the treatment becomes futile); interrupting the treatment means letting that person become worse and even die, thus breaching the principle of non-maleficence.

If the physician receives another patient, he must explain to him the fact that his/her resources are limited, and that he might not receive a particular treatment another person has received, due to insufficient resources. The patient can accept this status quo or not, in which case he can seek another physician (or refuse treatment). The patient can, of course, always request the interruption of treatment (even a life-saving one), but only if he/she has insight. If not, neither the physician nor the legal representative should be allowed to interrupt the treatment unless there are clear signs of the patient’s wish (living will, notarized acts), or when the treatment becomes futile.

## Conclusion

Age should never be used as the sole criterion for resource allocation in the COVID-19 pandemic. Physicians should treat everybody who has a medical indication, even if the odds are against a favorable course of the disease and should stop only when they deem the treatment as futile. Statistics should only be used to provide objective, evidence-based criteria for establishing medical indications.

Age should never be used as a unique criterion for withholding/not initiating life-saving procedures, even in pandemics or cases in which healthcare resources are extremely scarce. This approach is based on fundamental Codes of Ethics, such as the WMA Code of Ethics or the Oath of Hippocrates, and all physicians actually treating patients should obey them.
